# Metabolic abnormalities in patients with prolactinoma: response to treatment with cabergoline

**DOI:** 10.1186/s13098-015-0094-4

**Published:** 2015-11-14

**Authors:** Nazir A. Pala, Bashir A. Laway, Raiz A. Misgar, Rayees A. Dar

**Affiliations:** Department of Endocrinology, Sher-i-Kashmir Institute of Medical Sciences, Soura, Srinagar, Jammu and Kashmir India; Department of Biostatics, Sher-i-Kashmir Institute of Medical Sciences, Soura, Srinagar, Jammu and Kashmir India

**Keywords:** Cabergoline, Hyperprolactinemia, Metabolic syndrome, Insulin resistance, Prolactin, Lipids

## Abstract

**Background:**

Hyperprolactinemia has been associated with changes in body composition and metabolic abnormalities. Normalization of prolactin (PRL) with dopamine agonists has been found to reverse these abnormalities. This study was designed to assess the anthropometric and metabolic alterations associated with prolactinoma and response of these abnormalities to cabergoline treatment.

**Methods:**

In a non-randomised matched prospective design, 19 consecutive patients with prolactinoma (median PRL 118.6 (105.3) μg/L) and 20 controls were studied. The controls were age, gender and body mass index (BMI) matched. Anthropometric data and metabolic variables were studied at baseline, 3 and 6 months after cabergoline treatment.

**Results:**

Patients with prolactinoma had increased level of fasting plasma glucose (*P* < .001), LDL-cholesterol (*P* = .001) and triglycerides (TG) (*P* = .009) as compared to age, gender and BMI matched healthy controls. There was a significant decrease of body weight at 3 months (*P* = .029), with a further decline at 6 months (*P* < .001) of cabergoline therapy. In addition, there was a significant decrement of BMI (*P* < .001), waist circumference (*P* = .003), waist-hip ratio (*P* = .03) and total body fat (*P* = .003) at 6 months of cabergoline treatment. A significant decline in plasma glucose (*P* < .001), total cholesterol (*P* = .009), LDL-cholesterol (*P* < .001) and TG (*P* < .001) was seen after 6 months of cabergoline treatment.

**Conclusions:**

Patients with prolactinoma have adverse metabolic profile compared with matched controls. Normalization of PRL with cabergoline corrects all the metabolic abnormalities.

## Background

Prolactin (PRL) exerts a wide variety of effects on metabolism, in addition to its well-known actions on lactation and gonadal function [1–4]. Several animal and few human studies (especially in men) describe a higher body weight in patients with hyperprolactinemia (HPL), the exact mechanism of which is not clear [5, 6]. A combination of factors like decreased dopaminergic tone, low adiponectin, hypogonadism with or without associated leptin resistance could contribute to weight gain [7–10]. Hyperprolactinemia has also been linked to alteration in glucose homeostasis, insulin sensitivity and lipid parameters. Hyperglycemia with or without insulin resistance has been demonstrated in men and women with HPL because of its effects on the growth of islet cells and insulin production [11, 12]. Many studies have demonstrated adverse lipid profiles such as increased low-density lipoprotein (LDL), triglycerides (TG) and decreased high-density lipoprotein (HDL) in people with HPL. This could be due to a decrease in lipoprotein lipase activity in fat cells and subsequent effects on adipose tissue [13]. Effects of normalization of PRL [with dopamine agonists (DA)] on body weight, glucose and lipid homeostasis is a matter of debate and is being explored [14–16]. There is some disagreement on metabolic abnormalities in people with HPL and so is its response to treatment with DA. Aims of the present research were: (1) to study the metabolic abnormalities in people with prolactinoma compared to healthy age, gender and body mass index (BMI) matched controls and (2) the effect of normalization of PRL with cabergoline treatment on these metabolic abnormalities.

## Methods

### Study population

This study was conducted in the Department of Endocrinology at a tertiary care hospital in North India over a period of 1½ years. Twenty-eight consecutive patients with prolactinoma because of PRL secreting pituitary adenoma and twenty age, gender and BMI matched controls, were included in the study. Out of 28 patients, eight women were excluded (four conceived and four were lost to follow up). One man with prolactinoma and hypogonadism, on testosterone replacement was also excluded in the final analysis. Thus, the final sample consisted of 19 patients (eighteen women and one man) and 20 controls. Controls were healthy first or 2nd degree relatives without any hypertension, diabetes and were not any medications. The study was approved by the Institutional Ethics Committee and informed consent was obtained from all patients and controls. Patients with elevated PRL levels on at least two occasions and magnetic resonance imaging (MRI) evidence of pituitary adenoma were included. Exclusion criteria were secondary causes of HPL, pregnancy or patients on medication for dyslipidemia and diabetes mellitus at study entry.

### Study protocol

Anthropometric and laboratory measurements in cases and controls were assessed at baseline. Patients with prolactinoma were reassessed at 3 and 6 months after initiation of cabergoline treatment. Blood samples were taken after an overnight fast for measurement of metabolic parameters (glucose and lipid profile), and hormones like insulin, PRL, thyroid stimulating hormone (TSH), thyroxine (T4), testosterone, growth hormone (GH), follicle stimulating hormone (FSH) and luteinizing hormone (LH). Insulin resistance was measured by using homeostasis model assessment-insulin resistance (HOMA-IR) which is calculated as a product of fasting plasma insulin (mIU/L) and glucose (mmol/l) divided by 22.5. All the patients were treated with cabergoline (0.5 mg orally/week) with dosage titration to normalise serum PRL. Serum PRL was measured monthly until normalization. During the study period, patients were instructed to continue their routine homemade diets and not to curtail their routine diet and activity.

### Anthropometric data

Both cases and controls were examined by measurement of weight and height, using standard measurements. BMI was calculated as weight in kgs divided by square of height in meters (kg/m^2^). Measurements of waist and hip were taken to calculate waist-hip ratio (WHR). Total body fat was measured by dual energy X-ray absorptiometry (DEXA) scan (GE lunar DPX pro) at baseline in both cases and controls and after 6 months of cabergoline treatment in patients with prolactinoma. Obesity was defined as per “Asia-Pacific guidelines of obesity classification” [17]. BMI of 23–24.9 kg/m^2^ was considered as overweight and ≥25 kg/m^2^ as obesity.

### Biochemical and hormonal assays

Serum PRL was measured using a commercial chemiluminescent immunoassay (Beckman Coulter Unicel, DXI) normal range: 1–27 μg/L (women); 1–20 μg/L (men). TSH, T4, FSH, LH, GH, cortisol and testosterone were also measured by using commercial chemiluminescent immunoassays (Beckman Coulter Unicel, DXI). Plasma glucose was estimated by enzymatic method using glucose oxidase and peroxidase on an automated chemistry analyzer (HITACHI-912). Lipid parameters were analysed with commercially available enzymatic reagents (Audit Diagnostics, Ireland) adapted to the Hitachi 912 autoanalyzer. The upper normal limit for reference population for fasting total cholesterol, LDL, HDL, TG and glucose were 5.18, 3.10, 1.8, 2.26 and 5.5 mmol/l respectively. Insulin was measured by immunoradiometric assay (Insulin IRMA, Beckman coulter, France) by using commercial kits with intra-assay and inter-assay coefficients of variation, 3.99 and 4.80 % respectively (normal reference 20.83–111.12 pmol/l).

### Statistical analysis

The statistical software SPSS 20 was used to analyse the data. The continuous variables are shown in terms of descriptive statistics like mean, SD, median and inter quartile range. The Parametric and non-parametric tests have been used to analyse the data after verifying the distribution of variables with the help of Shapiro–Wilk test. The Student’s independent t test and Wilcoxon-Mann–Whitney U test was used to compare the parameters between cases and controls at baseline. Also the paired t test and Wilcoxon signed rank test has been used to analyze the data at baseline and 3 and 6 months of cabergoline treatment. All results have been described on 5 % level of significance i.e. *P* value of <.05 being considered as significant.

## Results

Out of 19 patients, 15 had microadenoma and four had macroadenoma. Out of 18 women, one had primary amenorrhoea, 12 had secondary amenorrhoea and the other five were eumenorrheic. Four women had primary infertility and two had secondary infertility. Two women had primary hypothyroidism and were on adequate doses of levothyroxine at the time of entry in the study. Two patients with macroprolactinoma had morning cortisol <275.9 mmol/l. Insulin tolerance test demonstrated normal cortisol and GH responses in both of these patients. In none of these patients, was there any suggestion of extension of pituitary mass into third ventricle on MRI.

Serum PRL normalized in 18 out of 19 patients after 1 month and in one patient within 6 months of cabergoline therapy. Median PRL level decreased from 118.6 μg/L at baseline to 10.4 μg/L at 3 months (*P* < .001) and 9.4 μg/L at 6 months (*P* < .001).

### Metabolic parameters in cases and controls

Out of 19 patients, seven (36.8 %) were obese, four (21 %) were overweight and two had impaired fasting glucose. Among controls, four (20 %) were obese, four (20 %) were overweight and none had impaired fasting glucose. Mean (SD) age of patients was 27.3 (6.2) years and BMI was 24.2 (4) kg/m^2^. With cases and controls comparable in body weight and BMI, patients with prolactinoma had significantly higher trunk fat on DEXA measurement: [mean (SD) truncal fat of 11.0 (4.2) kg in cases and 9.3 (2.8) kg in controls (*P* = .044)], though there was no difference in total body fat. Patients with prolactinoma had significantly higher levels of fasting plasma glucose compared to controls (4.8 ± 0.5 mmol/l in cases and 4.2 ± 0.3 mmol/l for controls, *P* < .001). LDL-cholesterol was significantly higher in cases compared to controls (2.9 ± 0.8  vs 2.1 ± 0.5 mmol/l, *P* = .001). Similarly TG were significantly higher in cases compared to controls (1.8 ± 0.4 vs 1.4 ± 0.4 mmol/l, *P* = .009). HDL-cholesterol in cases tended to be lower than that of controls but did not achieve statistical significance (Table 1).

**Table 1:** Baseline characteristics of cases and controls

Parameter	Cases (n = 19)	Controls (n = 20)	*P* value
Age^a^	27.3 (6.2)	27.1 (5.6)	.9
Gender (men:women)	1:18	2:18	
Body weight (kg)^a^	57.3 (9.5)	60.3 (11.1)	.35
BMI (kg/m^2^)^a^	24.2 (4.0)	24.6 (3.9)	.73
Waist (cm)^a^	85.3 (12.0)	83.3 (7.4)	.52
Hip (cm)^a^	91.1 (8.7)	91.4 (9.0)	.91
Waist–hip ratio^a^	0.94 (0.12)	0.92 (0.09)	.68
Body fat (kg)	23.4 (10.4)	20.5 (8.3)	.69
Trunk fat (kg)^a^	11.0 (4.2)	9.3 (2.8)	.044
Fasting glucose (mmol/l)^a^	4.8 (0.5)	4.2 (0.3)	<.001
Fasting insulin (pmol/l)	34.3 (54.6)	35.9 (22.3)	.82
HOMA-IR	1.1 (1.27)	1.4 (0.59)	.64
Total cholesterol (mmol/l)	4.5 (1.4)	3.4 (1.6)	.02
LDL (mmol/l)^a^	2.9 (0.8)	2.1 (0.5)	.001
HDL (mmol/l)	1.1 (0.3)	1.2 (0.3)	.04
Triglycerides (mmol/l)^a^	1.8 (0.4)	1.4 (0.4)	.009
Uric acid (µmol/l)^a^	297.9 (66.6)	206.9 (75.5)	<.001
FSH (IU/l)^a^	5.7 (2.6)	5.8 (1.8)	.88
LH (IU/l)^a^	5.3 (4.4)	5.2 (1.5)	.96
Estradiol (pg/ml)^a^	52.7 (24.3)	85.7 (42.6)	.018

*HOMA-IR* homeostatic model of insulin resistance, *LDL* low density lipoprotein cholesterol, *HDL* high density lipoprotein cholesterol

^a^Normally distributed data are expressed as mean (SD). Other data (non-normally distributed) expressed as median (interquartile range)

### Metabolic parameters before and after cabergoline treatment

The prevalence of obesity decreased from 36.8 % at baseline to 21 % at 6 months after cabergoline treatment. There was a significant decline in mean (SD) body weight from 57.3 (9.5) kg at baseline to 54.8 (9.2) kg after 6 months (*P* < .001). Similarly, there was a significant decrease in mean (SD) BMI from 24.2 (4.0) kg/m^2^ at baseline to 23.2 (3.9) kg/m^2^ (*P* < .001) after 6 months of cabergoline treatment. There was a significant decrease in fasting plasma glucose from 4.8 ± 0.5–4.3 ± 0.3 mmol/l, LDL-cholesterol from 2.8 ± 0.9–2.0 ± 0.3 mmol/l and TG from 1.8 ± 0.4–1.2 ± 0.2 mmol/l after 6 months (*P* < .001 for all). Similarly, a trend was seen in total cholesterol levels falling from 4.5 ± 1.0 mmol/l at baseline to 3.6 ± 0.5 mmol/l (*P* = .009) after 6 months of treatment (Table 2). Two patients had impaired fasting glucose, which normalized after treatment. There was a trend towards an increase in insulin sensitivity, as is evident from a decrease in plasma glucose and HOMA-IR, after achievement of normoprolactinemia at 6 months of treatment (Table 2). After 6 months of cabergoline treatment, LDL- cholesterol decreased to a level comparable to controls whereas TG and plasma glucose showed a further decline.

**Table 2:** Anthropometric and hormonal profile of patients at diagnosis and after 3 and 6 months on cabergolin treatment

Parameter	Baseline	Cabergoline treatment		*P* value	
		3 months	6 months	*	†
Body weight (kg)^a^	57.3 (9.5)	56.6 (9.1)	54.8 (9.2)	.029	<.001
BMI (kg/m^2^)^a^	24.2 (4.0)	23.9 (4.2)	23.2 (3.9)	.09	<.001
Waist (cm)^a^	85.3 (12.0)	84.9 (11.3)	82.3 (10.0)	.30	.003
Waist-hip ratio^a^	0.9 (0.06)	0.9 (0.06)	0.9 (0.07)	.89	.039
Body fat (kg)	23.4. (10.4)	–	22.3 (11.8)	–	<.001
PRL (µg/L)	118.6 (105.3)	10.45 (20.0)	9.4 (5.9)	<.001	<.001
TSH (U/L)^a^	3.4 (1.7)	3.7 (2.0)	4.3 (1.3)	.60	.09
T4 (nmol/l)^a^	110.5 (25.2)	125.3 (33.9)	114.4 (22)	.10	.60
Fasting glucose (mmol/l)^a^	4.8 (0.5)	4.6 (0.4)	4.3 (0.3)	.006	<.001
Fasting insulin (pmol/L)	34.3 (54.6)	37.6 (43.3)	32.2 (20.9)	.62	.24
HOMA-IR	1.1 0 (1.27)	1.21 (1.10)	1.04 (0.52)	.71	.064
Total cholesterol (mmol/l)^a^	4.5 (1.0)	4.1 (0.8)	3.6 (0.5)	.11	.009
LDL-cholesterol (mmol/l)	2.8 (0.9)	2.3 (0.5)	2.0 (0.3)	.01	<.001
HDL-cholesterol (mmol/l)	1.1 (0.3)	1.0 (0.2)	1.1 (0.2)	.36	.32
Triglycerides (mmol/l)^a^	1.8 (0.4)	1.3 (0.4)	1.2 (0.2)	.004	<.001
FSH (IU/l)	6.0 (3.8)	5.6 (4.0)	5.6 (3.2)	.87	1.00
LH (IU/l)	5.1 (7.4)	5.23 (2.7)	5.3 (1.3)	.62	.54
Estradiol (pg/ml)^a^	52.7 (24.3)	87.7 (54.5)	63.4 (167.4)	.004	.12

*HOMA-IR* homeostatic model of insulin resistance

Baseline compared with * 3 months and ^†^6 months after cabergolin treatment

^a^Normally distributed data are shown as mean (SD). Other data (non-normally distributed) are expressed as median (interquartile range)

### Hormonal profile of cases and controls

Mean estradiol at baseline in patients with prolactinoma was significantly lower as compared to controls (52.7 ± 24.3 pg/ml in cases and 85.7 ± 42.6 pg/ml for controls, *P* = .018) and it increased to 87.7 ± 54.5 pg/ml after 3 months (*P* = .004) without any further significant increase after 6 months of cabergoline treatment (*P* = 0.12). Concentrations of FSH and LH were in normal range both at beginning and at the end of treatment.

To understand whether the improvement in metabolic parameters was because of a decrease in weight after cabergoline treatment, patients were sub grouped into two categories, one with a decrease and another without change in BMI after cabergoline treatment. We found that there was significant change in fasting plasma glucose, LDL-cholesterol and TG at 3 and 6 months of cabergoline therapy in both the groups.

## Discussion

The metabolic consequences of HPL are being explored. HPL has been found to be associated with increased food intake and weight gain which promotes obesity [5, 7]. In line with the hypothesis of a pivotal role of dopaminergic tone in regulating body weight, the decrease in BMI and body fat content following PRL normalization in hyperprolactinemic patients receiving treatment with cabergoline has been ascribed to type 2 dopamine receptor activation [18].

### Metabolic abnormalities in prolactinoma

Many metabolic abnormalities have been described in men and women with HPL. Patients with HPL have insulin resistance and glucose intolerance compared to normal individuals [19, 20]. In the present study, we documented higher levels of fasting plasma glucose in patients with prolactinoma compared to age, gender and weight matched controls. Many previous case control studies have documented higher insulin resistance and abnormal glucose tolerance in patients with prolactinoma compared to healthy controls [21–24]. In the present study, fasting plasma insulin and glucose were high in patients with prolactinoma compared to age, gender and BMI matched healthy controls implying that diabetogenic effect of HPL may be independent of gender and body weight. We documented increased levels of LDL-cholesterol and TG in patients with prolactinoma compared to age, gender and BMI matched healthy controls. We also documented high total cholesterol and low HDL in patients with prolactinoma compared to controls but the difference was not statistically significant. The association between HPL and hyperlipidemia (especially increased total cholesterol and TG) has been documented as early as 1982 in a case control study [25]. In a subsequent case control study, low HDL-cholesterol has been documented though there was no difference in serum TG and LDL-cholesterol [26]. In another study, total cholesterol and LDL-cholesterol concentration was found to be higher in women with hyperprolactinemic amenorrhea compared to age matched controls [27]. The abnormal lipid profile in people with HPL is contributed partly by increase in body weight and partly because of the lipogenic action of PRL [28]. Taking a BMI matched control population; we did not demonstrate any increase in body fat on DEXA, though trunk fat was significantly higher in patients with prolactinoma. There is a limited data on body fat distribution in patients with HPL. Some studies have revealed a high body fat mass in patients with HPL [21]. Patients with HPL had lower estradiol concentrations as compared to that of controls, which is related to hypogonadism.

### Effect of normalization of prolactin with cabergoline on metabolic parameters

In patients with HPL, the effect of DA treatment on body weight and other metabolic parameters is a subject of active research. Some, but not all the studies have documented reduction in body weight and BMI in patients with prolactinoma after treatment with DA [19–23]. The effect of DA treatment on weight seems to have gender difference. A recent study has shown that reduction in body weight and BMI after DA treatment is seen only in men [24], while another study revealed major reduction in body weight and BMI in men as compared to women [7]. In the present study, we documented significant reduction in body weight after 3 months with a further decline after 6 months of cabergoline treatment. We also documented significant reduction in BMI, WHR and body fat after 6 months of cabergoline treatment. Our study population predominantly involved women; therefore, the beneficial effects of DA treatment on weight may not be restricted to men only. HPL of any etiology has been found to be associated with weight gain [24, 29, 30]. Weight gain associated with HPL is believed to be because of changes in the appetite and satiety regulating systems [31, 32]. Dopamine agonists have been found to induce weight loss [31–34]. The improvement in weight and abdominal obesity after DA treatment may appear as early as 3 months as seen in the present study and persist even up to 5 years [24, 29]. We documented significant reduction of fasting plasma glucose after 3 months of cabergoline treatment with a further decline after 6 months of treatment.

There is recent evidence that insulin sensitivity improves after 6 months of cabergoline treatment as documented by improvement in HOMA-IR [26, 27]. In the present study, we documented improvement in HOMA-IR after 6 months of cabergoline treatment. Numerous studies have demonstrated an improvement in insulin sensitivity with DA in patients with HPL and changes are independent of variation in body weight [21, 23, 27, 35]. Though some short-term studies involving treatment of HPL with DA up to 12 weeks did not reveal any changes in insulin resistance and blood glucose [20], many studies have demonstrated significant improvement in insulin resistance after 6 months to 5 years of DA treatment. In addition, significant improvement in insulin sensitivity is documented in adult obese individuals without HPL after treatment with bromocriptine [36].

In the present study, we documented significant reduction of LDL-cholesterol and TG after 3 months of cabergoline treatment with a further decline at 6 months. We also documented significant reduction of total cholesterol after 6 months (but not at 3 months) of treatment. Many studies have shown improvement in lipid profile after treatment with DA. The improvement seen can be as early as 2 months and up to 5 years of treatment [19, 31, 35]. The changes in lipid parameters are seen well before the changes in the body weight suggesting that improvement may be because of direct effect of DA and not related to weight. Also, in the present study, there was a significant decrease in plasma glucose, LDL and TG after 3 and 6 months after treatment with cabergoline, independent of change in body weight. Treatment with bromocriptine has been shown to decrease total cholesterol in obese non-diabetic hyperinsulinemic women [37]. Continued improvement in lipid parameters has also been shown with higher doses of cabergoline even after 12 months of treatment [38]. In the present study, after completion of 6 months of cabergoline treatment, fasting plasma glucose, LDL-cholesterol and TG in cases were similar to that of controls indicating reversal of these metabolic abnormalities. The fall in TG and plasma glucose was well below the level seen in age and BMI matched controls suggesting that cabergoline may have a role in decreasing TG beyond PRL decreasing effect. After 3 months of cabergoline treatment, there was a significant increase in estradiol as compared to baseline without any further increase after 6 months.

### Limitations of the study

Two important limitations of the present study include small sample size and short follow-up.

Further studies with a larger cohort and longer duration of follow-up are required, perhaps including long-term cardiovascular outcomes as well.

## Conclusion

The current study demonstrated that, patients with prolactinoma have metabolic abnormalities like increased truncal fat and high levels of plasma glucose, LDL-cholesterol, TG and lower levels of estradiol compared to healthy controls. We demonstrated a significant reduction of body weight, BMI, waist, WHR, total body fat, fasting plasma glucose, total cholesterol, LDL-cholesterol, TG with a significant increase in estradiol after treatment with cabergoline, with total cholesterol and TG decreasing to lower levels compared to matched healthy controls. Insulin sensitivity improved but did not achieve a statistical significance. We suggest that it is important to consider the metabolic profile of patients with prolactinoma before a decision to stop DA treatment is taken.

## Abbreviations

BMI: body mass index
DA: dopamine agonists
DEXA: dual energy X-ray absorptiometry
FSH: follicle stimulating hormone
GH: growth hormone
HDL: high density lipoprotein
HPL: hyperprolactinemia
HOMA-IR: homeostasis model assessment for insulin resistance
IRMA: immuno-radiometric assay
LDL: low density lipoprotein
LH: luteinizing hormone
MRI: magnetic resonance imaging
PRL: prolactin
SD: standard deviation
TG: triglycerides
T4: thyroxine
TSH: thyroid-stimulating hormone
WHR: waist-hip ratio
